# Using Optical Tweezers to Characterize Physical Tethers at Membrane Contact Sites: Grab It, Pull It, Set It Free?

**DOI:** 10.3389/fcell.2016.00022

**Published:** 2016-03-29

**Authors:** Imogen Sparkes

**Affiliations:** Biosciences, College of Life and Environmental Sciences, University of ExeterExeter, UK

**Keywords:** membrane contact sites, tether, optical tweezer, MCS, biophysics

Compartmentalisation is a defining feature of eukaryotic life. Effective communication between organelles is essential for cell maintenance, growth and response to external stimuli. Static snapshots provided through ultrastructural studies of preserved tissue highlight that certain organelles are in intimate contact at membrane contact sites (MCSs), also referred to as inter-organellar tethering sites. However, live cell imaging indicates that these interactions are not necessarily stable with organelles frequently “colliding,” moving in unison and then separating. This dramatic intracellular “waltz” between organelles with ever changing partners (organelles) indicates that the molecular factors controlling MCSs are highly regulated. Key questions therefore relate to defining which organelles physically interact, deciphering the molecular components that control MCS formation, and ultimately deciphering the specific functional role that the interaction provides to the cell (Figure 1).

**Figure 1:**
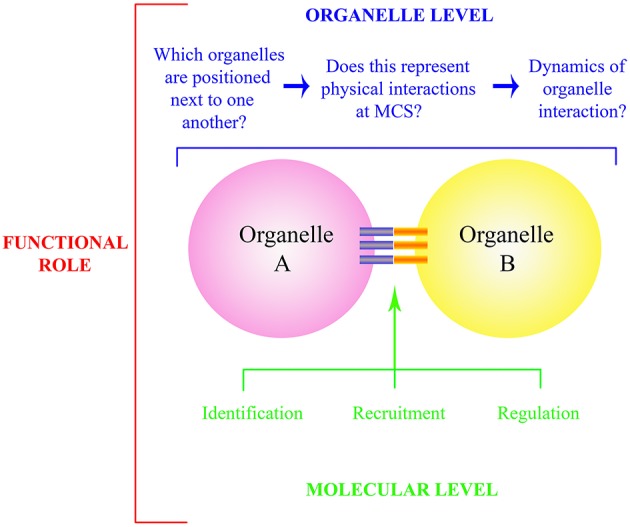
**Schematic representation of questions relating to organelle interactions and MCS**. The functional role of organelle interaction (red) is determined at the whole organelle (blue) and molecular level (green). At the organelle level, *bona fide* physical interactions need to be identified, and the regulation of such is governed through identification, recruitment and regulation of the molecular components that comprise the MCS itself (blue and orange bars). These components include both physical tethers and those which allow exchange at the MCS.

Reviews on the roles of MCSs are covered elsewhere (Elbaz and Schuldiner, 2011; Helle et al., 2013; Prinz, 2014; Islinger et al., 2015; Phillips and Voeltz, 2016). Readers are also directed to the Frontiers special topic (Schrader and Islinger, 2016) and the special issue of Current Opinion in Cell Biology dedicated to cell organelles including MCSs (Schuldiner and Guo, 2015). Here, I will provide an overview of the techniques used to interrogate MCSs and how optical tweezers could provide a future platform for characterizing the biophysical nature of MCSs.

Tethers have been isolated using multiple techniques; however a difficulty has been being able to discriminate between a role in physical tethering versus a role in transferring components at the MCS itself. For example, tethering sites are required for signaling, trafficking and biogenesis. Proteins located at MCSs could provide physical stability and MCSs formation, whereas others could collocate to and function in the actual transfer of molecules such as lipids and calcium. These generic roles are not necessarily mutually exclusive as evidenced by studies with OSBP (Mesmin et al., 2013). One way to discriminate between these two generic roles is if disruption of the potential tether affects the subsequent physical association between organelles. A clear example of this is observed during division of budding yeast where tethering can play a role in organelle inheritance into the bud cell. Dependent on the organism and cell type, organelles can be highly motile during interphase. Here, organelles are not clearly partitioned in a similar manner during cell division, and so seemingly random motion could result in organelles occupying similar physical regions without it being as a result of direct physical interaction *per se*. Organelle movement in higher plants is even further complicated by fast cytoplasmic streaming events.

Attempts to isolate and probe the nature of physical tethers include biochemical fractionation, genetic screens and microscopy. Applications of these techniques to investigate MCSs are covered in more depth in the review by Helle et al. (2013).

Biochemical fractionation and concentration of membrane enriched fractions have resulted in mitochondrial associated membranes (MAMs), plasma membrane associated membranes (PAMs) and plastid associated membranes (PLAMs). Here, enriched fractions highlight differential lipid and protein content between opposing organelle membranes and an intermediary fraction which contains components from both organelle membranes. The latter is thought to represent MCS enriched regions. In principle, this is a straight forward process, however in practice, identification and characterization of the molecular factors that are enriched in these fractions, can be problematic (e.g., reconciling subcellular location with function at MCS).

Novel ways to isolate tethers have included a synthetic screen in yeast which pulled out the ER-mitochondria tethering complex, ERMES (Kornmann et al., 2009). Tethering components have also been isolated using more traditional genetic screens. For example, components of the Store-Operated Calcium Entry (SOCE) at the ER-PM were isolated through independent RNAi screens (Liou et al., 2005; Roos et al., 2005; Feske et al., 2006; Vig et al., 2006; Zhang et al., 2006).

Transmission electron microscopy (TEM) allows ultra-structural observations of MCSs between organelles to be quantified in terms of the number and length of the MCS, and the distance between the interacting organelles. Conventional analysis of single TEM images limits interpretation of organelle interactions to the plane that has been sectioned. Therefore, relatively large numbers of sections need to be quantified to provide statistically robust conclusions on the number of MCSs. TEM tomography however determines spatial relatedness through analysing serial sections of a fixed sample, or by tilting the block *in situ*, to provide a three dimensional overview of the intimate association and connections between organelles. Examples of the use include determination of the interaction between the ER and mitochondria, and endosomes in yeast (Friedman et al., 2011; Alpy et al., 2013).

Visualization of MCS in dynamic living tissue is difficult. They typically bridge a gap of up to 30 nm between organelles, and so are below the limit of resolution of conventional light microscopy. However, a clear advantage over EM and tomography is that the tissue is live allowing dynamic events to be observed, and also negates any potential artifacts that may have been introduced during the fixation procedure. Technological advances in imaging are now beginning to combine the advantageous properties of live cell imaging with the ultra-structural resolution offered through EM. By breaking the diffraction limit of light, and using algorithms to compute spatial positioning and relatedness between imaged structures, super resolution light microscopes can provide enhanced spatial resolution with sufficient scan speeds to capture organelle movement; for example STORM and RESOLFT systems have been used to image ER dynamics (Grotjohann et al., 2012; Shim et al., 2012). These imaging systems are not commonplace, and the dynamic range may not capture fast movement events. Traditionally, conventional light microscopy has been used to quantify organelle movement and correlate movement patterns of organelles which appear closely associated and / or move in tandem. The open question here is whether this reflects true physical association of the two organelles, coordinated movement through co-regulated motors or organelles that are traversing the same cytoskeletal track in a densely packed cytoplasmic environment?

The techniques highlighted above (biochemical fractionation, genetic screens and microscopy) cover certain aspects of MCS research. However, none of these techniques directly probe the biophysical nature of organelle interaction. Spatial relatedness could be caused by many reasons, not just through the role of the tethering process itself. For example, decreasing the cytoplasmic volume for organelles to occupy could result in increased “interactions” through mere random collisions of the organelles in a more highly constrained region, perhaps even changes in cytoplasmic viscosity may artificially elevate observed interactions through sheer issues of physically moving the organelles through a more viscous medium. Biophysical techniques which allow the user to physically “pull” apart organelle pairings *in vivo* are therefore advantageous.

Optical tweezers allows the user to physically trap an object which has a significantly different refractive index to the surrounding media, in this case the organelle in the cytoplasm. The trapped organelle can then be micromanipulated and moved laterally within the cytoplasm and interactions with neighboring organelles interrogated; Is more force required to move an organelle if it is next to a certain organelle indicating physically interaction? How does the interaction change in response to altering the properties of the tethers themselves?

Optical tweezers have been used to trap and move Golgi bodies in Arabidopsis leaf epidermal cells (Sparkes et al., 2009). This qualitative approach highlighted that movement of trapped Golgi, in turn remodeled the ER indicating a physical association between the two organelles. Furthermore, observations of the remodeled ER indicated that it could be “hooked” or anchored in place at regions within the cell, indicative of anchoring to the plasma membrane (PM). Further studies have highlighted the molecular components involved in the ER-PM sites, with the sites themselves being implicated in mechanosensing (Wang et al., 2014; Perez-Sancho et al., 2015). Contacts between the chloroplast and ER also appeared to occur in laser ablated Arabidopsis protoplasts and pea leaves (Andersson et al., 2007).

More recently, Gao et al. (2016) developed a quantifiable platform for using optical tweezers to measure organelle interactions in intact cells, more specifically the interaction between peroxisomes and chloroplasts. Here, using an automated platform to trap a peroxisome, users moved it a set distance at a set speed, and then monitored and quantified the effects on the organelle during this process; was it trapped? Did it stay in the trap during the lateral automated motion at a set speed? How did these characteristics vary with changes in optical trap strength? Was more force required to move and separate a peroxisome from a neighboring chloroplast? Based on these observations at low optical trap strength peroxisomes either escaped the trap during the lateral movement or were not trapped at all. As optical trap strength increased the percentage of trapped organelles increased with a concomitant decrease in organelles that escaped the trap or could not be trapped. These characteristics for two populations of peroxisomes, which were either next to a chloroplast or far away from a chloroplast, were monitored and compared. Results indicated that it was physically “harder” to trap and move chloroplast associated peroxisomes compared to those that were not associated with chloroplasts, indicative of a tethering mechanism between the two compartments. By doing this type of quantitative analysis, and making comparisons between juxtaposed organelles and control measurements of organelles which are not near one another, provides a clear indication of physical interaction between the two compartments. Furthermore, by monitoring the movement of the peroxisomes after turning the trap off, the authors were able to model the motion which relates to the tethering process itself.

It is also worth noting an alternative biophysical approach to quantifying organelle interactions. By using a femtosecond laser to generate a pressure wave within the cell, users can estimate the force required to effectively move or “push” an organelle. This is quite different to optical tweezers which uses submicron precision to specifically “pull” rather than “push” an organelle. Both approaches have been used to establish physical connections between peroxisomes and chloroplasts (Oikawa et al., 2015; Gao et al., 2016).

Optical tweezers can therefore be used to monitor and probe physical interactions between organelles. By using a quantifiable platform (such as that descried by Gao et al.) it also has the potential to interrogate the role of molecular components that drive the interaction itself. Here, one might expect that tethering efficiency at the MCS may be affected upon altered tether expression; overexpression may increase tethering, whereas mutations in the tether could determine the functional domains / critical residues required to maintain the physical interaction between organelles. Quantification of interactions in this way is laborious, and so it is not advisable to attempt a genetic screen to identify novel tethers using an optical tweezer strategy. Similar to its use in measuring force values exerted by molecular motors *in vivo* (for example Hendricks et al., 2012, PNAS; Rai et al., 2013, Cell), optical tweezers could also quantify the forces involved in organelle interactions.

The future of MCS research will be shaped through a combination of several complementary techniques. By understanding the limitations and advantages that each technical approach provides, users will break through the barriers in understanding MCS structure and regulation. It will be interesting to see if technological advances will allow multiple techniques to be combined into the one modular system to allow attributes of individual MCSs to be probed simultaneously. For example, being able to measure the dynamics of interactions between components of the tether complex, whilst ascertaining the force imparted by the interactions to maintain spatial positioning of the organelles. Our basic picture of eukaryotic life consisting of discrete membrane bound compartments is certainly being challenged by MCS studies. One looks forward to seeing the results from future endeavors in deciphering this layer of subcellular complexity.

## Author contributions

The author confirms being the sole contributor of this work and approved it for publication.

### Conflict of interest statement

The author declares that the research was conducted in the absence of any commercial or financial relationships that could be construed as a potential conflict of interest.
